# Association between serum γ-glutamyl transferase and advanced colorectal adenoma among inpatients: a case-control study

**DOI:** 10.3389/fonc.2023.1188017

**Published:** 2024-01-12

**Authors:** Huijie Wang, Huanwei Zheng, Xu Cao, Ping Meng, Jinli Liu, Haiying Zuo, Teng Zhang, Zhichao Wang

**Affiliations:** ^1^Department of Endoscopy, Shijiazhuang Traditional Chinese Medicine Hospital, Shijiazhuang, China; ^2^Department of Gastroenterology, Shijiazhuang Traditional Chinese Medicine Hospital, Shijiazhuang, China; ^3^Graduate School, Hebei North University, Zhangjiakou, China; ^4^Institute of Traditional Chinese Medicine, North China University of Science and Technology, Tangshan, China

**Keywords:** advanced colorectal adenoma, serum γ-glutamyl transferase, association, retrospective study, case-control study

## Abstract

Emerging evidence suggests a link between γ-glutamyl transferase (GGT) and various malignancies. However, the relationship between GGT and advanced colorectal adenoma, a critical precursor to colorectal cancer, remains unclear. This study aimed to elucidate this relationship. We conducted a single-center retrospective study from April 2015 to June 2022, enrolling 3534 inpatients including 525 cases and 3009 controls. Data were extracted from the electronic medical records, encompassing clinicodemographic characteristics, co-morbidities, and several blood biochemical indicators. Utilizing logistic regression and curve fitting, we explored the relationship between GGT and advanced colorectal adenoma. After adjustment for confounding factors, we found that for each 20-unit increase in GGT, the risk of advanced colorectal adenoma increased by 6% (OR= 1.06 [1.01–1.12]). Moreover, individuals with high GGT levels (≥50 U/L) had a 61% higher risk of advanced colorectal adenoma compared to those with low GGT levels (<50 U/L) (OR=1.61 [1.13–2.31]). Subgroup analysis demonstrated the robustness of these findings across subjects with different characteristics. High GGT levels were associated with higher odds of advanced colorectal adenoma. Our findings suggest that elevated GGT levels may serve as a potential diagnostic marker for advanced colorectal adenoma, providing new insights into its screening strategies.

## 1 Introduction

Colorectal cancer (CRC) is considered a marker of socioeconomic development, with its incidence often rising with the increase of the Human Development Index in countries experiencing major transitions (1, 2). GLOBOCAN 2020 indicates that providing cancer prevention and care in countries in transition like China is critical to global cancer control (3). The incidence of CRC in China is becoming similar to that in countries with high HDI, but its CRC mortality is higher than in many developed countries. One possible explanation is that late-stage CRC tends to be prevalent in China (4, 5). This highlights the importance of early diagnosis and intervention of CRC. CRC is primarily caused by the slow progression of precancerous lesions (such as adenoma). The colorectal adenoma is a proliferative dysplastic epithelial lesion that is harmful in most cases. Depending on their size, number, histology (degree of dysplasia), and duration, they may undergo malignant transformation (6). Moreover, advanced adenoma is the direct precursor lesion of CRC (7), and early identification and treatment of patients with advanced adenoma remain the primary task for successful control and prevention of CRC.

γ-glutamyl transferase (GGT) is an enzyme anchored on the cell membrane, acting as a catalyst in the hydrolysis of gamma-glutamyl groups present in glutathione and glutathione-S-conjugates. At normal physiological concentrations, GGT is instrumental in maintaining intracellular equilibrium, metabolizing glutathione, and detoxifying foreign substances (8, 9). In clinical practice, GGT measurement is a routine diagnostic procedure, primarily recognized as a marker for hepatobiliary disorders and a biological indicator of excessive alcohol consumption (10). Recently, some research has sparked interest in elevated levels of GGT, observing a positive correlation between GGT and cancer risk, including respiratory cancer (11), gastrointestinal cancer (12–15), breast cancer (12, 16, 17), endometrial cancer (18), prostate cancer (12, 19), etc. Additionally, recent studies demonstrated a positive relationship between GGT levels and overall cancer incidence or mortality (20–22). Furthermore, the persistent reactive oxygen species (ROS) produced by GGT-mediated metabolic processes have been proven to participate in tumor development and invasion (23, 24). The discovery of increased GGT activity in malignant lesions indicates that GGT promotes rapid turnover and survival advantages of tumor cells (25). A review emphasized that elevated GGT levels could potentially heighten the likelihood of future cancer in the general population (22). We hypothesize that serum GGT levels are positively correlated with the risk of advanced adenoma, the most important precancerous lesion in the colorectal adenoma-cancer progression sequence.

Given that no studies have characterized the relationship between GGT levels and advanced colorectal adenoma, we will investigate the relationship between GGT and advanced colorectal adenoma, aiming to improve non-invasive screening programs for high-risk populations of advanced colorectal adenoma.

## 2 Methods

### 2.1 Study population

**Figure 1:**
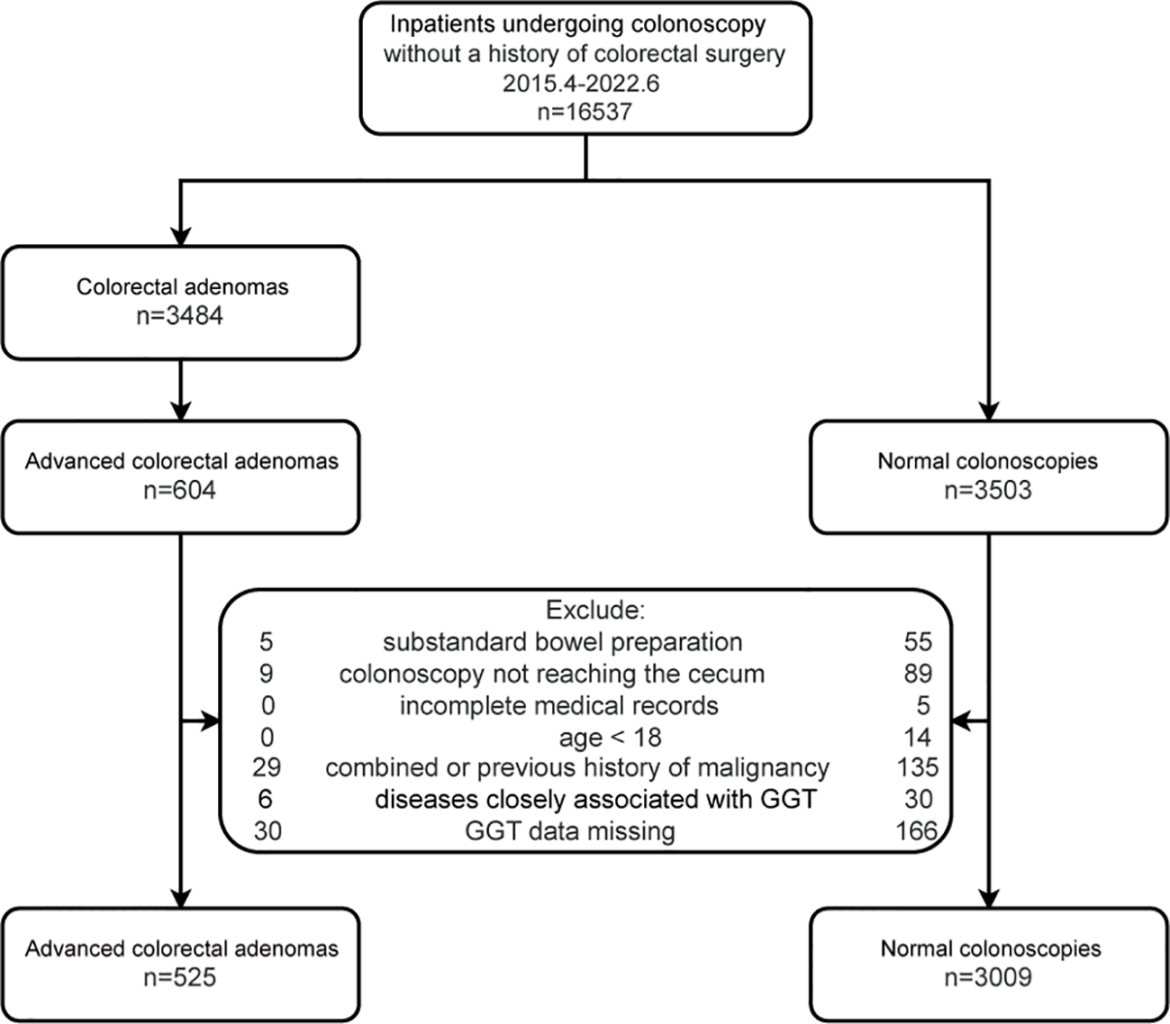
Flow diagram of participants.

Inpatients with advanced colorectal adenoma who were consecutively hospitalized from April 2015 to June 2022 were included as the case group. During the same period, all inpatients who were consecutively admitted for colonoscopy and had no colorectal diseases were considered as the control group. Exclusions were made for those with incomplete medical records, colonoscopy not reaching the cecum, inadequate bowel preparation, age less than 18 years, history of malignant tumors, diseases related to GGT (cholestasis, cirrhosis, drug-induced liver injury, nephrotic syndrome, pancreatitis, and pancreatic cancer), and missing GGT data. Each participant was included only once in this study. A total of 3534 subjects were analyzed in this study, which included 525 cases and 3009 controls. The flowchart is shown in **Figure 1**.

### 2.2 Definition of outcomes and indicators

Advanced colorectal adenoma was defined in this study as per the criteria established in the literature (26). Participants’ data were obtained from the electronic medical records, which included clinicodemographic characteristics and co-morbidities. Marital status was categorized as married, single/divorced, and others. Smoking and drinking status were classified as current, former, never, and NA (not available). A former smoker or former drinker was defined as someone who had previously smoked or consumed alcohol but had since ceased. Comorbidities included hyperlipidemia (HLP), coronary heart disease (CHD), ischemic cerebrovascular disease, hypertension, non-alcoholic fatty liver disease (NAFLD), and diabetes mellitus (DM).

Additionally, this study incorporated several laboratory tests, including total bilirubin (TBIL), direct bilirubin (DBIL), albumin (ALB), alanine aminotransferase (ALT), alkaline phosphatase (ALP), aspartate aminotransferase (AST), γ-glutamyl transferase (GGT), glucose, total bile acid (TBA), cholinesterase (ChE), uric acid (UA), creatinine (CREA), urea, total protein (TP), triglyceride (TG), total cholesterol (TC), high-density lipoprotein (HDL), low-density lipoprotein (LDL), apolipoprotein B, and lipoprotein(a). All laboratory test results used were the first results obtained before the colonoscopy during the hospitalization period.

### 2.3 Statistical analysis

We analyzed the baseline characteristics and co-morbidities of all participants. Continuous data were expressed as mean ± standard deviation or median (Q1–Q3), depending on the normality of the distribution. Group comparisons used the chi-squared or Fisher’s exact test for categorical variables, and Student’s t-test or the Mann-Whitney *U* test for continuous variables, as appropriate.

To account for missing data and minimize bias while maximizing statistical power, we conducted multiple imputations (5 replications). Sensitivity analyses were also conducted using complete cases.

The effect of GGT on the incidence of advanced colorectal adenoma was evaluated using binary logistic regression models (odd ratio [OR], 95% confidence interval [CI]). These models adjusted for major variables, including age, sex, drinking and smoking status, weight, marital status, ALT, TG, HDL, urea, UA, ALP, ALB, AST, TBIL, LDL, CREA, hypertension, DM, ischemic cerebrovascular disease, CHD. GGT levels were categorized into two groups: < 50 and ≥ 50 (cut-off point based on the reference range). We constructed four models: crude model; Model I, adjusting by age and sex; Model II, adjusting by Model I + weight, drinking status, smoking status, and marital status; Model III, adjusting by Model I + weight, drinking status, smoking status, hypertension, DM, ALB, ALT, TG, HDL, ALP, UA, AST, TBIL, urea, LDL, CREA, ischemic cerebrovascular disease, and CHD. Potential confounders were selected from significant covariates in the univariate analysis, prior scientific literature, or a change in effect estimate of more than 10%.

Stratified binary logistic regression models were conducted in subgroup analyses, followed by an interaction test. Due to the limited number of former smokers and former drinkers, these groups were merged with never-smokers and never-drinkers, respectively. Curve fitting was utilized to assess the association between the GGT (transformed using natural logarithm conversion) and advanced colorectal adenoma.

Statistical analyses were performed using R 3.3.2 (http://www.R-project.org, The R Foundation) and Free Statistics software version 1.7.1. A *P*-value of <0.05 was considered statistically significant (two-tailed).

## 3 Results

### 3.1 Baseline characteristics of participants

The study included 3534 participants. Detailed characteristics of the participants are presented in **Table 1**. On average, inpatients with advanced colorectal adenoma were older (mean age 61.4 ± 10.3), had a higher proportion of males (64.6%), and had a higher prevalence of comorbidities, including CHD, ischemic cerebrovascular disease, hypertension, and DM. They also exhibited higher levels of certain blood indicators, including ALP, GGT, TBA, glucose, UA, CREA, urea, and TG, and lower levels of TP, ALB, LDL, and HDL.

**Table 1:** Baseline characteristics.

Variables	Total n = 3534	Case n = 525	Control n = 3009	*P* value
Age, year	52.3 ± 13.0	61.4 ± 10.3	50.7 ± 12.8	**< 0.001**
Sex, male, n (%)	1456 (41.2)	339 (64.6)	1117 (37.1)	**< 0.001**
Weight, kg	67.1 ± 12.4	70.7 ± 11.8	66.5 ± 12.5	**< 0.001**
Marital status, n (%)				0.104
Single/divorced	152 (4.3)	14 (2.7)	138 (4.6)	
Married	3187 (90.2)	485 (92.4)	2702 (89.8)	
Others	195 (5.5)	26 (5)	169 (5.6)	
Drinking status, n (%)				< 0.001
Never	2229 (63.1)	299 (57)	1930 (64.1)	
Current	172 (4.9)	53 (10.1)	119 (4)	
Former	15 (0.4)	3 (0.6)	12 (0.4)	
NA	1118 (31.6)	170 (32.4)	948 (31.5)	
Smoking status, n (%)				< 0.001
Never	2238 (63.3)	297 (56.6)	1941 (64.5)	
Current	134 (3.8)	48 (9.1)	86 (2.9)	
Former	25 (0.7)	5 (1)	20 (0.7)	
NA	1137 (32.2)	175 (33.3)	962 (32)	
Family history, n (%)				
Digestive system malignancy	180 (5.1)	33 (6.3)	147 (4.9)	0.178
Colorectal cancer	49 (1.4)	10 (1.9)	39 (1.3)	0.271
TBIL, (μmol/L)	12.7 (9.8, 16.6)	12.9 (9.9, 16.7)	12.6 (9.8, 16.6)	0.632
DBIL, (μmol/L)	2.7 (2.0, 3.8)	2.9 (2.1, 3.8)	2.7 (2.0, 3.7)	0.051
TP, (g/L)	71.8 ± 5.3	70.7 ± 5.8	72.0 ± 5.2	**< 0.001**
ALB, (g/L)	44.3 ± 3.7	43.7 ± 4.1	44.4 ± 3.6	**< 0.001**
ALT, (U/L)	17.0 (13.0, 25.0)	18.0 (13.0, 25.0)	17.0 (12.9, 25.3)	0.086
AST, (U/L)	19.4 (16.7, 24.0)	19.0 (17.0, 23.0)	19.6 (16.4, 24.0)	0.914
GGT, (U/L)	19.0 (14.0, 28.0)	22.0 (16.0, 35.0)	18.0 (14.0, 27.0)	**< 0.001**
Cholinesterase, (U/L)	8887.9 ± 1939.3	8848.3 ± 1904.7	8894.9 ± 1945.5	0.612
ALP, (U/L)	74.8 ± 24.7	79.8 ± 25.2	74.0 ± 24.6	**< 0.001**
TBA, (μmol/L)	2.6 (1.4, 4.4)	2.9 (1.7, 5.1)	2.5 (1.4, 4.3)	**< 0.001**
Glucose, (mmol/L)	6.0 ± 1.7	6.4 ± 2.0	5.9 ± 1.7	**< 0.001**
UA, (μmol/L)	302.2 ± 94.3	322.7 ± 91.5	298.7 ± 94.3	**< 0.001**
Urea, (mmol/L)	4.8 ± 1.6	5.1 ± 1.9	4.7 ± 1.5	**< 0.001**
CREA, (μmol/L)	63.9 ± 21.9	71.7 ± 39.3	62.5 ± 16.8	**< 0.001**
TC, (mmol/L)	5.0 ± 1.0	4.8 ± 1.0	5.0 ± 1.0	**0.002**
TG, (mmol/L)	1.3 (0.9, 1.9)	1.5 (1.0, 2.1)	1.3 (0.9, 1.9)	**< 0.001**
LDL, (mmol/L)	2.9 ± 0.6	2.8 ± 0.7	2.9 ± 0.6	**0.009**
HDL, (mmol/L)	1.4 ± 0.3	1.3 ± 0.3	1.4 ± 0.3	**< 0.001**
Apo B, (g/L)	1.0 ± 0.2	0.9 ± 0.3	1.0 ± 0.2	0.161
Lp(a), (mg/L)	140.0 (75.5, 296.0)	149.0 (71.1, 308.0)	138.9 (76.0, 292.0)	0.512
Comorbidities, n (%)				
Ischemic cerebrovascular disease	364 (10.3)	69 (13.1)	295 (9.8)	**0.02**
Hypertension	875 (24.8)	218 (41.5)	657 (21.8)	**< 0.001**
Coronary heart disease	410 (11.6)	86 (16.4)	324 (10.8)	**< 0.001**
Hyperlipemia	346 (9.8)	54 (10.3)	292 (9.7)	0.679
Diabetes mellitus	598 (16.9)	147 (28)	451 (15)	**< 0.001**
Non-alcoholic fatty liver disease	220 (6.3)	27 (5.2)	193 (6.5)	0.268

Data presented are mean ± SD, median (Q1–Q3), or N (%). Bold values indicate statistical significance.

TBIL, total bilirubin; DBIL, direct bilirubin; TP, total protein; ALB, albumin; ALT, alanine aminotransferase; AST, aspartate aminotransferase; GGT, γ-glutamyl transferase; ALP, alkaline phosphatase; TBA, total bile acid; CREA, creatinine; UA, uric acid; TC, total cholesterol; TG, triglyceride; LDL, low-density lipoprotein; LDH, lactate dehydrogenase; HDL, high-density lipoprotein; Lp(a), lipoprotein(a); Apo B, apolipoprotein B; DM, diabetes mellitus; NA, not available.

### 3.2 GGT and advanced colorectal adenoma

Multivariable logistic regression analyses were used to evaluate the association between GGT (per 20 U/L) and advanced colorectal adenoma (**Table 2**). After adjusting for age and sex (Model I), GGT was positively associated with advanced colorectal adenoma (OR, 1.04 [1.01–1.07], *P*=0.007). The association remained stable after further adjustments in Model II (sex, age, weight, smoking status, drinking status, and marital status; OR, 1.04 [1.01–1.07], *P*=0.022) and Model III (Model I + TBIL, ALB, ALT, AST, ALP, CREA, UA, TG, LDL, HDL, urea, ischemic cerebrovascular disease, CHD, hypertension, and DM; OR, 1.06 [1.01–1.12], *P*=0.012), the relationship between GGT and advanced colorectal adenoma remained stable.

**Table 2:** Multivariable logistic regression analyses of serum γ-glutamyl transferase and advanced colorectal adenoma.

Variable, U/L	Event, N (%)	Crude model		Model I		Model II		Model III	
		OR (95% CI)	*P* value	OR (95% CI)	*P* value	OR (95% CI)	*P* value	OR (95% CI)	*P* value
GGT, per 20 unit	525/3534 (14.9)	1.05 (1.02~1.09)	0.001	1.04 (1.01~1.07)	0.007	1.04 (1.01~1.07)	0.022	1.06 (1.01~1.12)	0.012
GGT<50	455/3202 (14.2)	1(Reference)		1(Reference)		1(Reference)		1(Reference)	
GGT≥50	70/332 (21.1)	1.61 (1.22~2.14)	0.001	1.76 (1.29~2.4)	<0.001	1.56 (1.14~2.15)	0.006	1.61 (1.13~2.31)	0.009

GGT, γ-glutamyl transferase; TBIL, total bilirubin; ALB, albumin; ALT, alanine aminotransferase; AST, aspartate aminotransferase; ALP, alkaline phosphatase; CREA, creatinine; UA, uric acid; TG, triglyceride; LDL, low-density lipoprotein; HDL, high-density lipoprotein; CHD, coronary heart disease; DM, diabetes mellitus; OR, odds ratio; Cl, confidence interval.

Crude model: no other covariates were adjusted.

Model I: adjusted for sex and age.

Model II: adjusted for Model I + weight, smoking status, drinking status, and marital status.

Model III: adjusted for Model I + weight, smoking status, drinking status, TBIL, ALB, ALT, AST, ALP, CREA, UA, TG, LDL, HDL, urea, ischemic cerebrovascular disease, CHD, hypertension, and DM.

When GGT was used as a categorical variable, after adjusting for potential confounders in Model II, inpatients with high GGT levels (≥50 U/L) had a 56% increased risk of advanced colorectal adenoma compared to those with low GGT levels (<50 U/L) (OR, 1.56; 95% CI, 1.14–2.15; *P*=0.006). This association was further confirmed in Model III, where the risk was found to be 61% higher in the high GGT group (OR, 1.61; 95% CI, 1.13-2.31; *P*=0.009) (**Table 2**). The results indicate that elevated GGT levels may be an independent risk factor for advanced colorectal adenoma.

**Figure 2:**
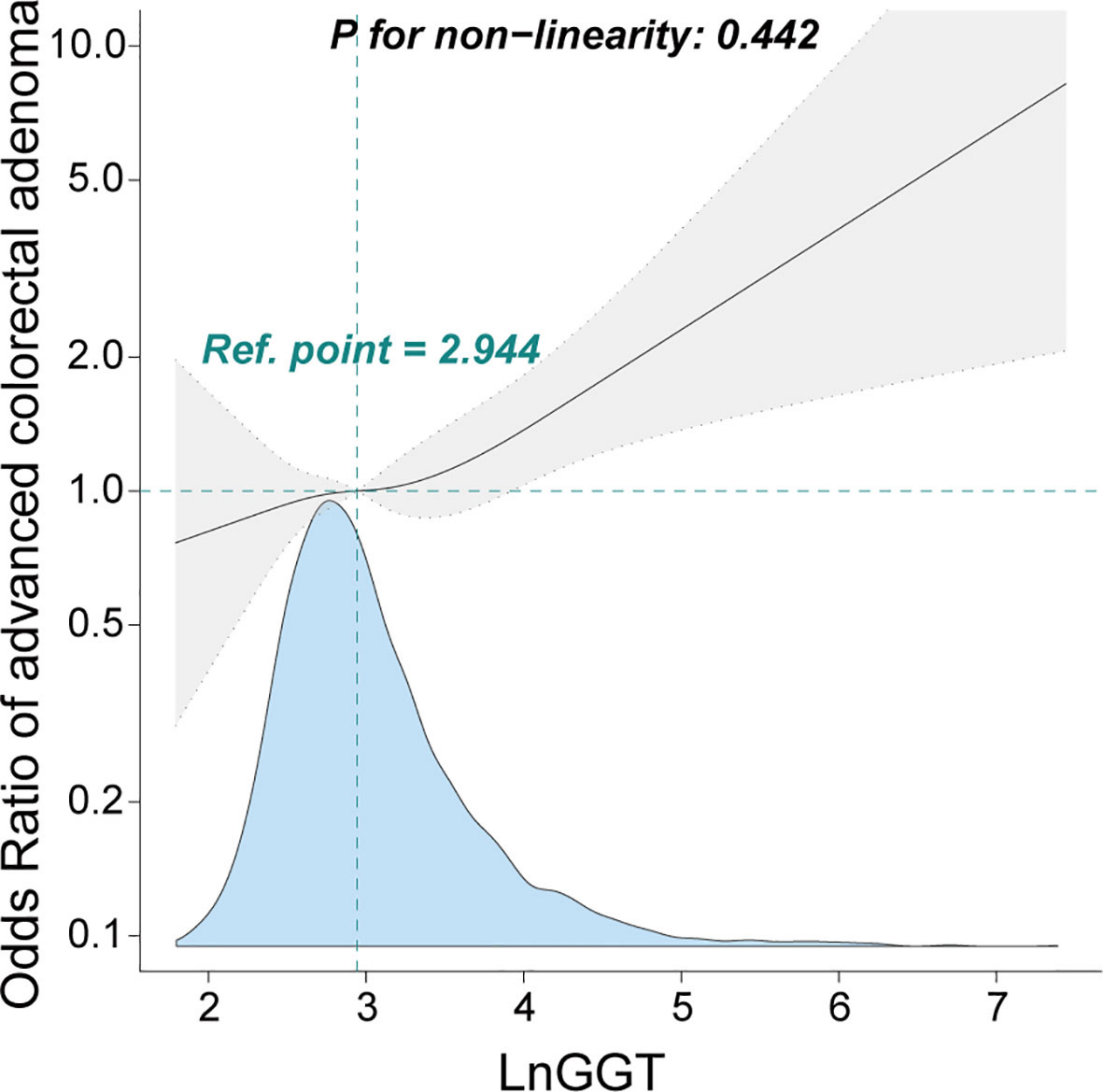
Association between γ-glutamyl transferase and advanced colorectal adenoma among inpatients. Odds ratio (OR) was adjusted for age, sex, smoking status, drinking status, weight, alanine aminotransferase, aspartate aminotransferase, alkaline phosphatase, total bilirubin, albumin, creatinine, uric acid, triglyceride, high-density lipoprotein, low-density lipoprotein, urea, ischemic cerebrovascular disease, diabetes mellitus, hypertension, and coronary heart disease. The γ-glutamyl transferase level was transformed using natural logarithm conversion.

Furthermore, the smooth curve analysis revealed a linear relationship between GGT and advanced colorectal adenoma among inpatients, even after adjustments were made for Model III (**Figure 2**).

### 3.3 Subgroup analysis

We conducted stratified analyses to assess the stability of the association between gamma-glutamyl transferase (GGT) levels and advanced colorectal adenoma across various subgroups. These subgroups included sex, age (<65 and ≥65 years), smoking status, drinking status, NAFLD, DM, ischemic cerebrovascular disease, hypertension, and CHD.

**Figure 3:**
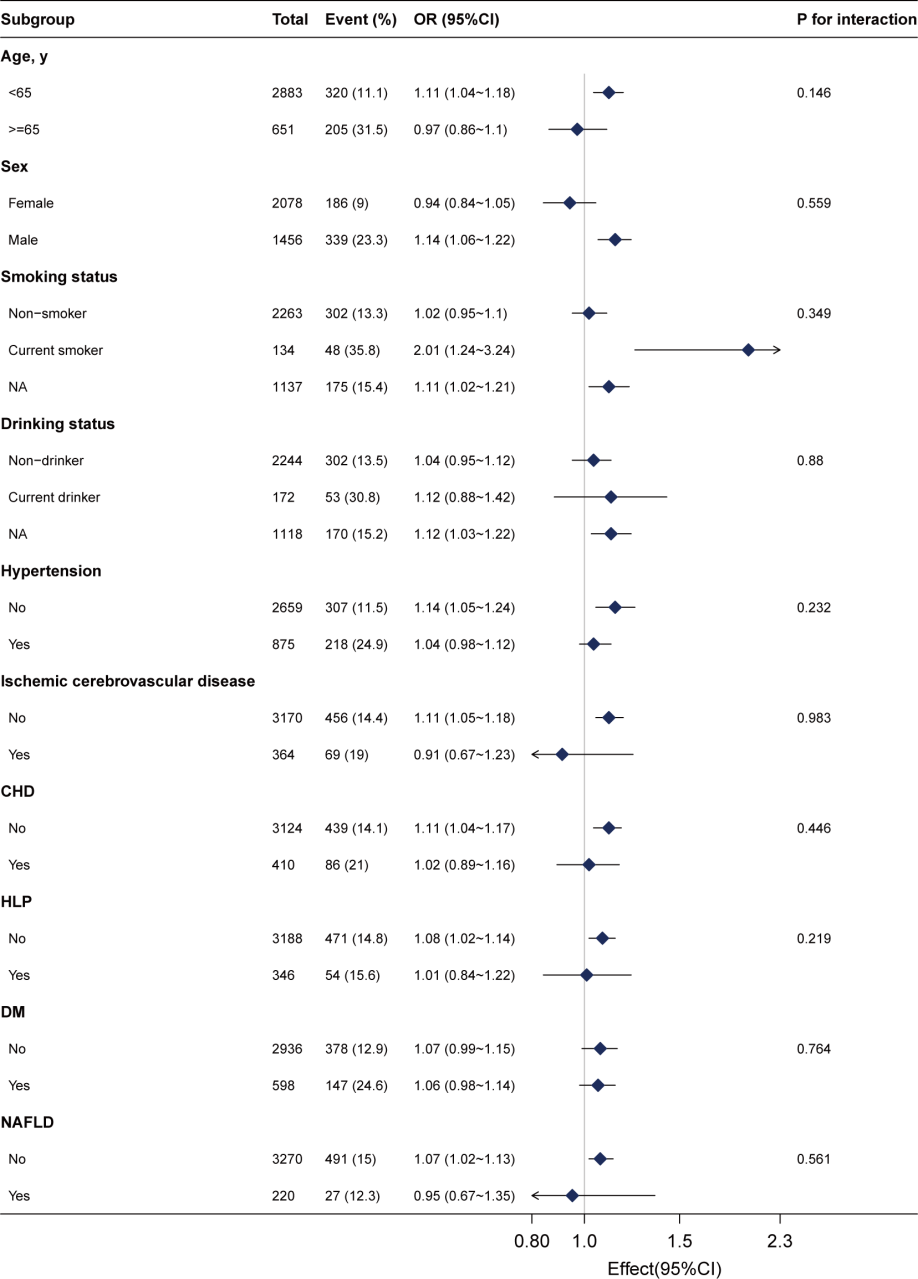
Subgroup analyses of the γ-glutamyl transferase (per 20 U/L) and advanced colorectal adenoma among inpatients. Each subgroup was adjusted for age, sex, weight, drinking status, smoking status, alanine aminotransferase, aspartate aminotransferase, alkaline phosphatase, total bilirubin, albumin, creatinine, uric acid, triglyceride, high-density lipoprotein, low-density lipoprotein, urea, ischemic cerebrovascular disease, diabetes mellitus, hypertension, and coronary heart disease.

After adjusting for sex, age, drinking status, smoking status, weight, ALB, ALT, AST, ALP, CREA, UA, TG, LDL, HDL, urea, CHD, ischemic cerebrovascular disease, hypertension, and DM, we found no significant interactions across these subgroups (**Figure 3**, all *P* for interaction >0.05). This suggests that the association between GGT and advanced colorectal adenoma is consistent across different demographic and clinical characteristics, further supporting the robustness of our findings.

### 3.4 Sensitivity analysis

In sensitivity analyses, we performed complete case analyses, excluding all participants with missing data. The results of these analyses, presented in **Supplementary Table S1**, were consistent with our primary findings, further validating our results.

In multivariable logistic regression analyses, GGT levels (per 20 U/L increase) were found to be positively associated with advanced colorectal adenoma across all models (Model I: OR, 1.04; 95% CI, 1.01–1.08; *P*=0.011; Model II: OR, 1.04; 95% CI, 1–1.07; *P*=0.034; Model III: OR, 1.07; 95% CI, 1.01–1.13; *P*=0.015).

When we categorized GGT levels, we found that participants in the high GGT group (≥50 U/L) had a 57% higher risk of advanced colorectal adenoma compared to those in the low GGT group (<50 U/L) (Model III: OR, 1.57; 95% CI, 1.04–2.37; *P*=0.033).

These sensitivity analyses, which account for potential bias due to missing data, provide additional support for the robustness of our findings.

## 4 Discussion

This study demonstrated a stable linear relationship between GGT and advanced colorectal adenoma.

Previous basic and clinical studies have indicated elevated GGT activity in the colorectal adenoma-carcinoma sequence. For instance, an animal study conducted in 1988 showed that the increase in GGT activity during carcinogenesis occurred early and was widespread. Rats injected weekly with dimethylhydrazine for a total of 16 weeks had significantly elevated GGT levels at 4 weeks, adenomas at 20 weeks, and carcinomas at 28 weeks compared to the controls (27). The increase in GGT activity continued throughout the carcinogenesis process. Additionally, a study using a GGT activatable fluorescent probe for fluorescence imaging of colorectal tumors found that GGT level was upregulated in cancer cells (28). This present study provided the first evidence of the clinical study that increased GGT levels were related to advanced colorectal adenoma. Recent studies showed an association between higher levels of GGT with CRC or non-advanced colorectal adenoma, even with GGT levels within the normal level. A systematic review and meta-analysis analyzed the published prospective evidence on the relationship between GGT and digestive cancer (1.94 [1.35–2.79]) (22). Elevated GGT levels were significantly associated with gastrointestinal cancer in a study based on the Korean national population (the highest quartile group, HR, 1.185 [1.158–1.211]) (15). In addition, a big-scale prospective study in Korea showed an association between high GGT levels and CRC in men (the highest quartile group, HR, 1.25 [1.15–1.36]) (13). Furthermore, a cross-sectional study demonstrated that elevated GGT may also be related to general colorectal adenoma with a cut-off point of >20 IU/L. Moreover, this study also found more prominent in patients without or with mild fatty liver (29). However, the relationship between GGT and advanced adenoma was stable in inpatients with or without NAFLD in our findings, probably attributable to the difference in the subjects. The present findings added to the evidence that advanced adenoma was also associated with elevated GGT, collectively suggesting that the entire progression of the colorectal adenoma-carcinoma sequence may be associated with elevated GGT.

Serum GGT has been extensively investigated as a diagnostic marker. In the adenoma-carcinoma sequence, Hong et al. explored the relationship between GGT levels and colorectal adenoma, employing the ROC curve and area under the curve (AUC) analyses (29). The AUC for GGT in predicting colorectal adenoma was 0.57, with a 95% CI of 0.55–0.60; the highest Youden index (1.125) corresponded to a cut-off value of ≥20 U/L, yielding a sensitivity of 0.63 and a specificity of 0.49. In a retrospective clinical study of 44 human diseases, Bai et al. assessed serum GGT levels as a biomarker for rectal and colon cancer using ROC curves, resulting in AUCs of only 0.53 and 0.51, respectively (30). Given these findings and considering the diagnostic performance of the fecal immunochemical test (FIT), currently the most commonly used screening tool for CRC (31), we posit that serum GGT may not be suitable as a standalone diagnostic marker for CRC in the adenoma-carcinoma sequence, as the diagnostic performance of FIT significantly surpasses that of GGT. However, the sensitivity of FIT in detecting adenoma and advanced adenoma is suboptimal, underscoring the urgent need for more accurate screening strategies to identify adenoma, particularly advanced adenoma. Considering the potential non-specificity of elevated serum GGT in patients with advanced adenoma, the combined use of GGT and other indicators as a composite diagnostic marker may offer improved diagnostic performance and potentially meet the screening requirements for advanced colorectal adenoma. To date, no studies have explored the diagnostic performance of GGT in different stages of CRC (including the advanced colorectal adenoma stage), representing a potential direction for future research.

The underlying mechanism between GGT and advanced colorectal adenoma is still to be investigated, however, our findings are probably biologically plausible based on the available evidence. Several hypotheses have been proposed for the positive association of GGT with the adenoma-carcinoma sequence. Based on a publicly available database, Lim et al. [26] found that as GGT increased, its activity maintained a negative correlation with six of the seven serum antioxidants, thus establishing a significant relationship between GGT levels and oxidative stress (32). It is commonly known that glutathione metabolism is the main thiol antioxidant in the body, and GGT is a resource of ROS during glutathione metabolism and is involved in mediating oxidative stress (33). ROS regulates key cellular functions such as proliferation, differentiation, growth, and apoptosis by acting as intracellular signaling molecules (34). CRC and adenoma stem from epithelial cells in the intestine. The rapid division and high metabolic rate of these cells have been identified as a potential contributor to increased DNA oxidation (35). Adenomatous polyps develop through the interplay between oxidative stress and involvement in colonic epithelial renewal and immune defense (36). Ros maintains and regenerates epithelial cells by generating signals for both proliferation and differentiation. Dysregulation of signaling in the intestinal epithelium can lead to the production of small lesions, called abnormal crypt foci, whose expansion leads to adenoma that can develop into carcinomas *in situ* and then into invasive adenocarcinomas (37). Overall, the increased GGT activity in epithelial lesions may lead to a sustained production of ROS induced by its mediated metabolism, bringing a rapid renewal and survival advantage to the lesioned cells.

In this cross-sectional study, although we adjusted for indicators reflecting liver function such as ALT, and confounding factors such as alcohol consumption in the logistic regression model, and excluded participants with diseases related to elevated GGT, the association between GGT and advanced colorectal adenoma may still be influenced by unavailable medication data of the participants and other unknown confounding factors. The specificity of the association between GGT and advanced colorectal adenoma can be enhanced by characterizing GGT expression in tumors using immunohistochemical staining or fluorescent probes. An enzyme-activated fluorescent probe, γ-Glutamyl hydroxymethyl rhodamine green (gGlu-HMRG), developed by Urano et al. (38), has demonstrated that GGT is overexpressed in a variety of malignant tumors such as ovarian cancer, breast cancer, brain cancer, lung cancer, colon cancer, and peritoneal cancer, as well as head and neck squamous cell carcinoma (13, 28, 39–43). Currently, there are no studies characterizing GGT expression in advanced colorectal adenoma tissues, which could be a direction for our future research.

Our study also had several limitations. Firstly, as with all retrospective observational studies, inherent limitations may affect the validity and reliability of the findings. However, we conducted sensitivity analyses to assess the robustness of the results and found them to be stable. Secondly, the absence of serial measurements for GGT may limit the precision in ascertaining its longitudinal levels and their association with advanced colorectal adenoma. Thirdly, despite performing multivariable logistic regression, residual confounding effects could not be entirely excluded. Finally, as our study was conducted in China, the generalizability of the findings to populations with different demographic characteristics may be limited.

## 5 Conclusion

In conclusion, a linear association was observed between GGT and the prevalence of advanced colorectal adenoma among inpatients in China. This study provides robust evidence of an association between elevated GGT and advanced colorectal adenoma, a relationship that remains consistent across subjects with diverse characteristics. Importantly, this study brings attention to this association, potentially offering a novel avenue for the early diagnosis of advanced colorectal adenoma.

## Data availability statement

The original contributions presented in the study are included in the article/**Supplementary Material**. Further inquiries can be directed to the corresponding author.

## Ethics statement

The studies involving humans were approved by the Ethics Review Board of Shijiazhuang Traditional Chinese Medicine Hospital. The studies were conducted in accordance with the local legislation and institutional requirements. The ethics committee/institutional review board waived the requirement of written informed consent for participation from the participants or the participants’ legal guardians/next of kin because equirements for informed consent were waived due to the retrospective nature.

## Author contributions

HW and HuZ conceived and designed the study. HW, XC, PM, JL, ZW, TZ, and HaZ collected and analyzed the data. HW and HuZ wrote and revised the manuscript. All authors contributed to the article and approved the submitted version.
