# Supplementary material for: Association between serum γ-glutamyl transferase and advanced colorectal adenoma among inpatients: a case-control study
Source: Front Oncol. 2024 Jan 12;13:1188017. doi: 10.3389/fonc.2023.1188017 (PMC10816217; doi:10.3389/fonc.2023.1188017)
Supplement: Supplementary file 1 [file Table_1.docx]

**Table S1** Multivariable logistic regression analyses of serum γ-glutamyl transferase and advanced colorectal adenoma (before multiple imputations, n=2656).

| Variable, U/L | Event,  N (%) | Crude model | |  | Model I | |  | Model II | |  | Model III | |
| --- | --- | --- | --- | --- | --- | --- | --- | --- | --- | --- | --- | --- |
|  |  | OR (95% CI) | *P* value |  | OR (95% CI) | *P* value |  | OR (95% CI) | *P* value |  | OR (95% CI) | *P* value |
| GGT, per 20 unit | 414/2656  (15.6) | 1.05 (1.01~1.09) | 0.008 |  | 1.04 (1.01~1.08) | 0.011 |  | 1.04 (1~1.07) | 0.034 |  | 1.07 (1.01~1.13) | 0.015 |
| GGT<50 | 360/2400  (15.0) | 1(Reference) |  |  | 1(Reference) |  |  | 1(Reference) |  |  | 1(Reference) |  |
| GGT≥50 | 54/256  (21.1) | 1.51 (1.1~2.09) | 0.011 |  | 1.73 (1.22~2.47) | 0.002 |  | 1.51 (1.05~2.18) | 0.026 |  | 1.57 (1.04~2.37) | 0.033 |

Abbreviations: GGT, γ-glutamyl transferase; TBIL, total bilirubin; ALB, albumin; ALT, alanine aminotransferase; AST, aspartate aminotransferase; ALP, alkaline phosphatase; CREA, creatinine; UA, uric acid; TG, triglyceride; LDL, low-density lipoprotein; HDL, high-density lipoprotein; CHD, coronary heart disease; DM, diabetes mellitus; OR, odds ratio; Cl, confidence interval.

Crude model: no other covariates were adjusted.

Model I: adjusted for sex and age.

Model II: adjusted for sex, age, weight, smoking status, drinking status, and marital status.

Model III: adjusted for sex, age, weight, smoking status, drinking status, TBIL, ALB, ALT, AST, ALP, CREA, UA, TG, LDL, HDL, urea, ischemic cerebrovascular disease, CHD, hypertension, and DM.
